# Cerebral Microcirculatory Blood Flow Dynamics During Rest and a Continuous Motor Task

**DOI:** 10.3389/fphys.2019.01355

**Published:** 2019-10-24

**Authors:** Martin Müller, Mareike Österreich

**Affiliations:** Neurovascular Laboratory, Neurocenter, Lucerne Kantonsspital, Lucerne, Switzerland

**Keywords:** cerebral autoregulation, cerebral microcirculation, motor task, evoked flow, near-infrared spectroscopy, transcranial Doppler

## Abstract

**Objectives:** To examine the brain’s microcirculatory response over the course of a continuous 5-min elbow movement task in order to estimate its potential role in grading vaso-neural coupling compared to the macrocirculatory response.

**Methods:** We simultaneously recorded cerebral blood flow velocity (CBFV), changes in oxygenated/deoxygenated hemoglobin concentrations ([oxHb], [deoxHb]), blood pressure (BP), and end-tidal CO_2_ over 5-min periods of rest and left elbow movements in 24 healthy persons (13 women and 11 men of mean age ± SD, 38 ± 11 years). A low frequency range (0.07–0.15 Hz) was used for analysis by transfer function estimates of phase and gain.

**Results:** Elbow movement led to a small BP increase (mean BP at rest 83 mm Hg, at movement 87; *p* < 0.01) and a small ETCO_2_ decrease (at rest 44.6 mm Hg, at movement 41.7 mm Hg; *p* < 0.01). Further, it increased BP-[oxHb] phase from 55° (both sides) to 74° (right; *p* < 0.05)/69° (left; *p* < 0.05), and BP-[deoxHb] phase from 264° (right)/270° (left) to 288° (right; *p* < 0.05)/297° (left; *p* = 0.09). The cerebral mean transit time at 0.1 Hz of 5.6 s of rest remained unchanged by movement. Elbow movement significantly decreased BP-CBFV gain on both sides, and BP-CBFV phase only on the right side (*p* = 0.05).

**Conclusion:** Elbow movement leads to an increased time delay between BP and [oxHb]/[deoxHb] while leaving the cerebral mean transit time unchanged. Phase shifting is usually the more robust parameter when using a transfer function to estimate dynamic cerebral autoregulation; phase shifting at the microcirculatory level seems to be a better marker of VNC-induced changes than phase shifting between BP and CBFV.

## Introduction

Considering its pathophysiologic relevance to high blood pressure (BP), microangiopathic diseases, mild cognitive impairment, and dementia, the examination of cerebral microcirculation has been gaining widespread research interest (Birns et al., 2009; Pantoni, 2010; Marmarelis et al., 2014, 2017; Duncombe et al., 2017; de Heus et al., 2018; Müller et al., 2019). In addition to morphological changes, as demonstrated by computerized tomography or magnetic resonance imaging (MRI), regulation of the microcirculatory blood supply is believed to be a relevant part of disease progression (Birns et al., 2009; Pantoni, 2010; Duncombe et al., 2017; Müller et al., 2019). Near-infrared spectroscopy (NIRS) offers the opportunity to observe oxygenated and deoxygenated hemoglobin concentration changes ([oxHb], [deoxHb]) in the microcirculation of the brain’s upper cortical layer with a relatively high temporal resolution (Murkin and Arango, 2009). Their dynamic interplay with BP or cerebral blood flow velocity (CBFV) may potentially represent an early disease marker. While much research has been carried out on the interplay between BP and transcranial Doppler-derived CBFV for assessing cerebrovascular dynamics (for a comprehensive overview, see Claassen et al., 2016), the usefulness of NIRS parameters as a substitute for CBFV in this context has, surprisingly, not yet been well evaluated.

Cerebral blood flow (CBF) is the combined result of BP, metabolic, and neuronal regulatory influences. In recent years, the influence of vaso-neural coupling (VNC) on CBF was determined to be a relevant contributor which can be disturbed even when cerebral autoregulation remains intact (Salinet et al., 2013, 2015; Caldas et al., 2018). To assess the dynamics of CA (dCA), applying a transfer function analysis (TFA) on the relationship between BP and CBFV is the most frequently used approach nowadays. When applied over 5-min recording periods, the phase and gain results are reliable, and phase is more robust than gain for the grading of CA capabilities (Claassen et al., 2016; Sanders et al., 2018). Most VNC studies used short task periods (≤60 s) to investigate VNC (Chance et al., 1993; Obrig et al., 2000; Salinet et al., 2013, 2015; Caldas et al., 2018), and are, therefore, unsuitable for assessing TFA (Claassen et al., 2016). In addition to the TFA of the BP-CBFV macrocirculatory relationship, the TF analyses of the dynamics of the interaction between BP and [oxHb], as well as of [deoxHb] (microcirculatory response), have also shown that it is possible to grade CA failure (Reinhard et al., 2006; Cheng et al., 2012; Elting et al., 2018). The motivation behind and the hypothesis of our present study is that a long-lasting stimulus in a normal CA state shows a TFA response (either in the macrocirculatory or in the microcirculatory response) that can be used to grade VNC. Since the final aim of our study is to assess whether alterations in this VNC response interfere with patient’s daily life activities or rehabilitation capabilities, further studies on patients using this setup could not be justified if this hypothesis were to turn out not to be true.

## Materials and Methods

The study was approved by the Ethics Committee of Northwest and Central Switzerland and abided by the tenets of the Declaration of Helsinki, using good standards of clinical practice. Written informed consent was obtained from all subjects [*n* = 24; 13 women, 11 men; mean age ± SD (standard deviation): 38 ± 11 years (range 24–62 years)].

### Experimental Setting and Instrumentation

All investigations were performed in the late morning with subjects in a supine position with their head elevated at approximately 30° in a dimly lit room. Coffee or tea was last ingested a minimum of 4 h before the beginning of the assessments. Subjects were mainly right-handed (20 of 25) and all were non-smokers. Participants were carefully instructed to flex and extend their elbow over the full range of movement (= one movement cycle) at a pace of 1 Hz, as signaled by the beep of a computerized metronome. Before mounting the probes, subjects practiced performing several elbow movement cycles according to the metronome’s pacing.

We assessed microcirculation using the NIRO-200NX NIRS device (Hamamatsu Photonics, Herrsching, Germany). This NIRS device emits infrared light at three frequencies (735, 810, and 850 nm). After absorption by oxygenated and deoxygenated hemoglobin, the backscattered light has a different intensity. The differences in light intensities between the emitted and backscattered lights are correlated with the concentration of oxygenated and deoxygenated hemoglobin in the brain’s upper layers. We used self-adhesive NIRS probes in which the light emitting diode (LED) and the detecting photodiode were fixed, separated by a distance of 4 cm. The detecting probe was placed at the lateral frontotemporal skull to ensure it was above the vascular territory of the middle cerebral artery (MCA), and the emitting probe was placed 4 cm away at the frontal skull. After initial adjustments to determine the baseline hemoglobin concentration (in μmol/L), the NIRS device provides continuous percentage changes in [oxHb] and [deoxHb].

Although we mainly focused on NIRS, because the BP-CBFV system is a more well-established method of assessing cerebrovascular dynamics, and because we wanted to interpret the BP-[oxHb] and BP-[deoxHb] findings in light of the best established method, we also recorded CBFV. After fixing the NIRS probes, a head holder provided by the manufacturer of the TCD device (MultidopX, DWL; Compumedics, Sipplingen, Germany) was placed for the TCD examination. Both MCAs were identified using common criteria, and the TCD probes (2 MHz) were positioned with the help of the head holder. End-tidal CO_2_ (ETCO_2_) was measured *via* a small nostril tube connected to a capnograph embedded in the TCD device. BP was measured by finger plethysmography (Finometer Pro; Finapres Medical Systems, Amsterdam, The Netherlands) placed at the fingertip of the right index finger, with special attention paid to calibrating it to the pressure of the brachial artery. The BP signal was imported into the TCD device and simultaneously recorded along with CBFV and ETCO_2_.

After all probes were placed and the subject familiarized him- or herself with the surroundings and experimental setting, we started with a 5-min baseline recording period. Subsequently, the left elbow movement was performed at the 1-Hz frequency set by the computerized metronome. The BP measurement instrument was placed on the fingertip of the right index finger such that the right arm could not be moved.

### Data Preparation and Analysis

CBFV, BP, and ETCO_2_ data were collected at 100 Hz and NIRS data were collected at 5 Hz. The data were analyzed using Matlab (2018b, Math Works Inc., Natick, MA, USA). The data were visually inspected for artifacts, and only artifact-free 5-min periods of data were used. After aligning the time series with their common starting time point, each raw data time series was resampled by averaging over a 1-s interval.

These new data segments then underwent transfer function analysis (TFA). The coherence and TFA estimates of phase (in °) and gain between the different time series were extracted from their respective power auto spectra or cross spectra using Welch’s averaged periodogram method with a Hann window length of 100 s, a window overlap of 50%, and a total Fast Fourier Transformation data length of 300 s, thereby allowing for calculations over a frequency range of 0.02–0.40 Hz.

Because CA is mostly regulated in the frequency range of 0.07–0.15 Hz, and coherence has been shown to be low in the frequency range of 0.02–0.07 when [oxHb] and [deoxHb] are involved (Müller et al., 2016), we decided to only use the 0.07–0.15-Hz frequency range for this analysis. To calculate means, we averaged phase and gain values with a coherence ≥ 0.34 (Claassen et al., 2016). When large phase wrap-arounds were present in the phase spectrum plots, we subsequently corrected phase by adding or subtracting 360° (as recommended in Claassen et al., 2016). All analyses were primarily performed using the phase spectrum. When necessary for comparing with previously published literature, phases were transformed to their time domains. Cerebral mean transit times were calculated as phase (BP-[deoxHb]) − phase (BP-[oxHb]).

For each period, we also calculated cerebrovascular resistance as mean BP/mean CBFV, in addition to the heart rate derived from the BP signal.

### Statistical Analysis

The Matlab Statistical Toolbox was used for all data analyses. Using a Kolmogorov–Smirnov test, all data were shown to have a normal distribution, and the data are reported as mean ± standard deviation (SD). To compare means between the two periods (baseline and elbow movement), we used a one-way ANOVA, including, when indicated, a correction for multiple comparisons. A regression analysis was used to test whether the results were dependent on ETCO_2_, BP variability (as calculated as the SD of BP), cerebrovascular resistance (BP/CBFV), or BP-derived heart rate. Values of *p* ≤ 0.05 were considered statistically significant.

## Results

Artifact-free recordings were acquired bilaterally in all subjects. The hemodynamic baseline characteristics, including ETCO_2_, are reported in Table 1. Elbow movement induced significant increases in BP, heart rate, CBFV, [oxHB], and CVR (on the right side only), and a significant decrease in ETCO_2_ and [deoxHb].

**Table 1:** Hemodynamic baseline variables (mean ± standard deviation) at rest and during elbow movement over 5-min periods at a frequency of 1 Hz.

	Baseline	Elbow movement	ANOVA
Mean BP (mm HG)	83 ± 13	87 ± 17	<0.01
BP variability (mm HG)	16 ± 2	17 ± 2	ns
Mean ETCO_2_ (mm HG)	44.6 ± 3.0	41.7 ± 3.4	<0.01
Mean heart rate (beats/min)	65 ± 9	74 ± 11	<0.01
Mean CBFV right (cm/s)	65 ± 12	69 ± 12	<0.01
Mean CBFV left (cm/s)	64 ± 11	70 ± 13	<0.01
Mean CVR right	1.35 ± 0.29	1.28 ± 0.28	<0.01
Mean CVR left	1.32 ± 0.31	1.31 ± 0.32	ns
Mean [oxHb]r (%)	0.08 ± 0.11	0.55 ± 0.11	<0.001
Mean [oxHb]l (%)	−0.001 ± 0.08	0.19 ± 0.09	<0.001
Mean [deoxHb]r (%)	−0.56 ± 0.04	−0.81 ± 0.11	<0.001
Mean [deoxHb]l (%)	−0.60 ± 0.05	−0.84 ± 0.07	<0.001

*BP, blood pressure; ETCO_2_, end-tidal carbon dioxide; CBFV, cerebral blood flow velocity; [oxHb], oxygenated hemoglobin; [deoxHb], deoxygenated hemoglobin; r, right; l, left*.

**Figure 1:**
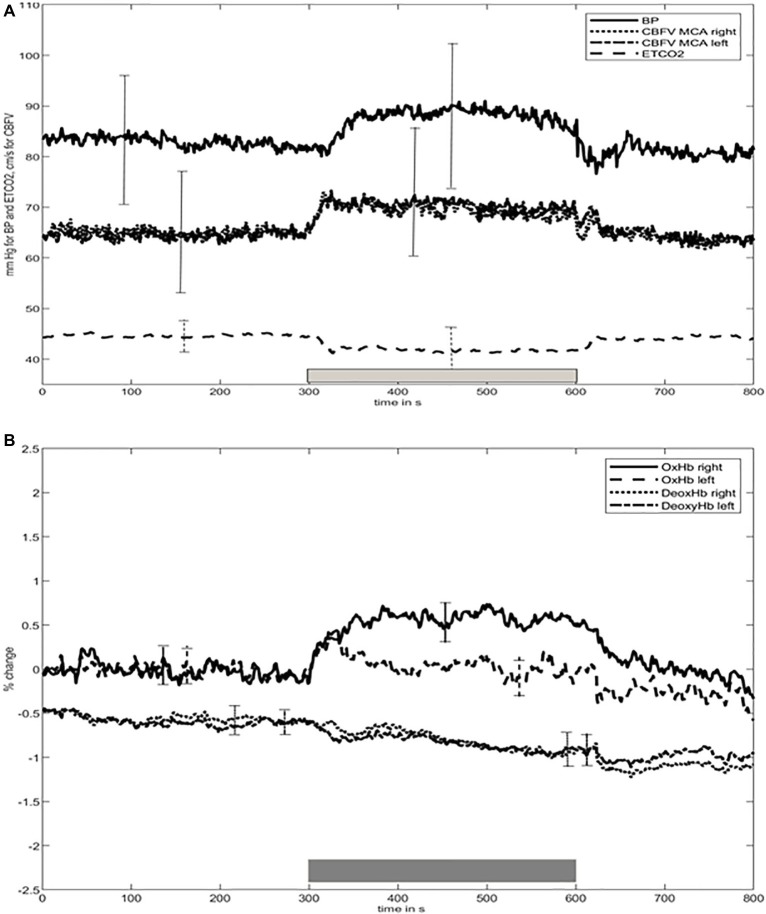
Time course of **(A)** blood pressure (BP), cerebral blood flow velocity (CBFV), end-tidal carbon dioxide (ETCO_2_), **(B)** concentration of oxygenated ([oxHb]) and deoxygenated ([deoxHb]) hemoglobin during baseline at rest and elbow movement time periods. BP, blood pressure; CBFV, cerebral blood flow velocity; ETCO_2_, end-tidal carbon dioxide; [oxHb], oxygenated hemoglobin; [deoxHb], deoxygenated hemoglobin. Bars indicate the standard deviation over all values of each time period. For convenience, since the SD bars of CBFV are nearly identical for both sides, we decided to only show one for both sides. The thick gray bar above the X-axis represents the timing of the elbow movement.

The time course of the group average means of BP, ETCO_2_, CBFV, [oxHb], and [deoxHb] is shown in Figure 1. Changes in CBFV, [oxHb], and [deoxHb] start very shortly after beginning the motor task. ETCO_2_ decreased 5 s thereafter, and, of note, BP rose afterward, after about 20 s. Subsequently, all variables remained largely constant until the end of the stimulus period. Mean [oxHb] changes over the total stimulus period were significantly higher in the right compared to left side (*p* < 0.001).

TFA results (Table 2) demonstrate, under resting conditions, a phase lead of CBFV to BP (mean ≈ −36°) and a time lag of [oxHb] (≈55°) and [deoxHb] (≈266°) behind BP. [oxHb] was 44° behind CBFV, and [deoxHb] 275° behind CBFV.

**Table 2:** Transfer function estimates (phase and gain) for blood pressure and cerebral blood flow velocity-dependent hemodynamic pairs (mean ± standard deviation).

	Baseline	Elbow movement	ANOVA
**BP-dependent system**		
Phase (in degrees)			
BP-CBFVr	−36 ± 10	−43 ± 9	*p* = 0.05
BP-CBFVl	−37 ± 13	−40 ± 17	ns
BP-[oxHb]r	55 ± 27	76 ± 34	*p* < 0.05
BP-[oxHb]l	55 ± 28	69 ± 31	*p* < 0.05
BP-[deoxHb]r	264 ± 38	288 ± 31	*p* < 0.05
BP-[deoxHb]l	270 ± 47	297 ± 33	*p* = 0.09
**Gain (in %/mm Hg)**		
BP-CBFVr	0.97 ± 0.35	0.73 ± 0.38	*p* < 0.01
BP CBFVl	1.01 ± 0.44	0.76 ± 0.31	*p* < 0.01
BP-[oxHbr]	0.005 ± 0.01	0.002 ± 0.01	ns
BP-[oxHb]l	0.007 ± 0.01	0.007 ± 0.03	ns
BP-[deoxHb]r	−0.003 ± 0.005	−0.001 ± 0.007	ns
BP-[deoxHb]l	−0.003 ± 0.005	−0.002 ± 0.01	ns
**CBFV-dependent system**		
Phase (in degrees)			
CBVF-[oxHb]r	47 ± 27	61 ± 23	*p* = 0.06
CBFV-[oxHb]l	46 ± 25	52 ± 35	ns
CBFV-[deoxHb]r	279 ± 41	261 ± 48	ns
CBFV-[deoxHb]l	273 ± 20	269 ± 37	ns
Gain (in %/cm/s)			
CBFV-[oxHb]r	0.007 ± 0.03	0.009 ± 0.01	ns
CBFV-[oxHb]l	0.002 ± 0.04	0.008 ± 0.03	ns
CBFV-[deoxHb]r	−0.003 ± 0.02	−0.002 ± 0.01	ns
CBFV-[deoxHb]l	−0.009 ± 0.03	−0.004 ± 0.02	ns
**Mean transit time (in degrees)**		
Right	209 ± 49	216 ± 33	ns
Left	201 ± 46	226 ± 53	ns

*BP, blood pressure; CBFV, cerebral blood flow velocity; [oxHb], oxygenated hemoglobin concentration; [deoxHb], deoxygenated hemoglobin concentration; r, right; l, left; mean transit time calculated as (BP-[HHb]) − (BP-[O_2_Hb])*.

**Figure 2:**
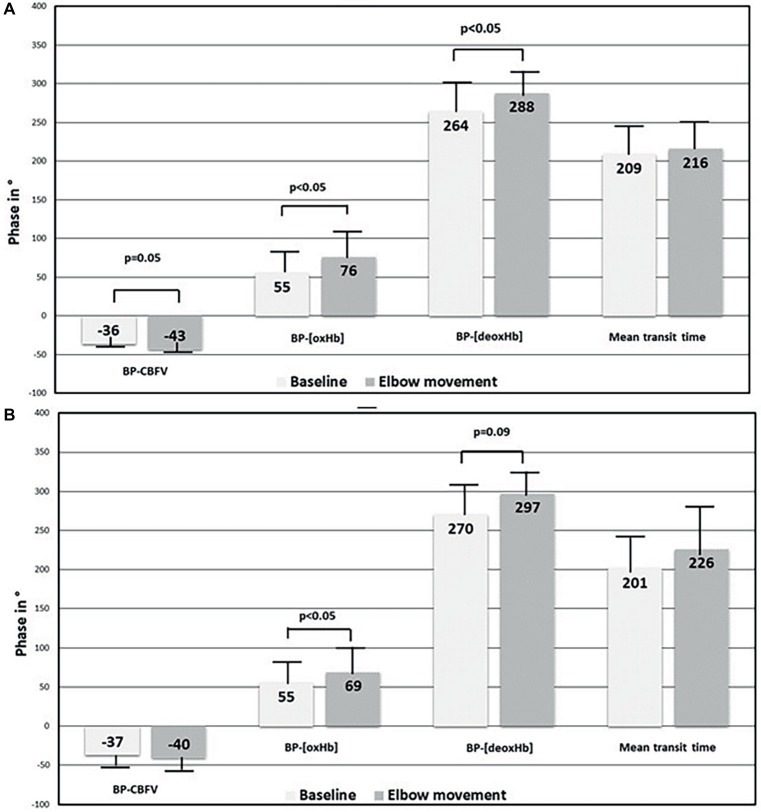
Comparison of phase shift results between baseline at rest and elbow movement in macrocirculation and microcirculation. **(A)** Right side and **(B)** left side. BP, blood pressure; CBFV, cerebral blood flow velocity; [oxHb], oxygenated hemoglobin concentration; [deoxHb], deoxygenated hemoglobin concentration.

Elbow movement induced several changes, among which the phase changes are summarized in Figure 2. First, elbow movement reduced the mean gain between BP and CBFV on both sides significantly from ≈1 to ≈0.75%/mmHg. The increases in the negative mean phase between BP and CBFV (from −36° (right)/−37° (left) to −40°/−43°; right n.s., left *p* = 0.05) were small. Second, elbow movement increased the positive mean phase shift between BP and [oxHb] from 55° (both sides) to 74° (right; *p* < 0.05)/69° (left; *p* < 0.05), and between BP and [deoxHb] from 264° (right)/270° (left) to 288° (right; *p* < 0.05) / 297° (left; *p* = 0.09). The mean transit time was not different between rest and elbow movement. Third, elbow movement increased the positive mean phase shift between CBFV and [oxHb] on both sides (from 47° to 62° on the right side, from 48° to 52° on the left side), which was relevant by trend (*p* = 0.06) on the right side.

On regression analysis, no TFA parameter was found to be related to heart rate, ETCO_2_, BP variability, or CVR, either under resting conditions, during elbow movement, or when combining both conditions.

## Discussion

In general, to assess the brain’s circulatory system by TFA, TFA is applied to the BP-CBFV relationship. When CA is intact, the CBFV phase precedes the BP phase. When CA is disturbed, this phase lead becomes smaller. This change in phase lead is variable, but when CA fails (for example by increasing blood CO_2_ partial pressure), it increases as a result of vasodilation (or loss of vessel stiffness). Our baseline findings related to the BP-CBFV relationship are consistent with those of previously published literature (Meel-van den Abeelen et al., 2014; Claassen et al., 2016; Sanders et al., 2018). If the TFA approach is applied to the microcirculation parameters [oxHb] and [deoxHb] and related to BP or CBFV as the driving (input) parameter, our knowledge about the human microcirculation’s dynamic remains limited. Gain varies widely, with inconsistent results. Under resting conditions, the differences in phase between BP or CBFV and [oxHb] or [deoxHb] range between 15 and 55° for BP-[oxHb], between 44 and 84° for CBFV-[oxHb], between 209 and 265° for BP-[deoxHb], and about 270° for the CBFV-[deoxHb] relationship (Reinhard et al., 2006; Cheng et al., 2012; Phillip et al., 2012; Fantini, 2014; Müller et al., 2016; Elting et al., 2018). The phase shift differences depend mostly on the mathematical approach used, data preparation, and the use of either spontaneous oscillatory CBF changes or enforced CBF changes by, for example, forced breathing (Reinhard et al., 2003, 2006; Cheng et al., 2012; Phillip et al., 2012; Müller et al., 2016; Elting et al., 2018). The reported mean transit times range between 190° (corresponding to 5.15 s at 0.1 Hz) in the investigation of Reinhard et al. (2006), 205° in our present investigation (corresponding to 5.6 s), and 120° (corresponding to 3.2 s) in Elting et al. (2018). Although these transit time differences appear large, they are in the normal range when cerebral blood flow studies using positron emission tomography or dynamic susceptibility contrast-enhanced MRI are considered for comparisons. For cortical or hemispheric blood flow, the transit time is reported to vary between 2 and 8 s, mainly around 4.3–5.1 s (Minns and Merrick, 1991; Ibaraki et al., 2007). Prolonged elbow movement led to several hemodynamic changes. It must be kept in mind that BP elevation and hypocapnia will lead to vasoconstriction (and an increased vessel stiffness) of the resistance vessels (Birch et al., 1995; Zhang et al., 1998, 2009; Panerai et al., 1999; Müller et al., 2003, 2019; Serrador et al., 2005), and a corresponding gain decrease in the TFA of the BP-CFBV relationship. In our work, the BP-CBFV relationship yielded a gain increase, indicating vasodilation despite vasoconstricting factors. Thus, from a macrocirculatory view, CBF increases at the macro- and the microcirculatory levels were induced by vasodilation, which would fit the increased metabolic demands. The demands were more prominent on the right side, explaining the stimulus-dependent side-to-side differences in [oxHb] (higher on the right side) and CVR (lower on the right side). At the microcirculatory level, forced breathing or hyperventilation increases phase shifting between BP and [oxHb] (Cheng et al., 2012). This finding is likely related to the macroangiopathic BP-CBFV changes in this condition (the BP-CBFV phase shift becomes more negative, meaning that BP is more delayed before entering intracranial circulation). Because our subjects hyperventilated, we consider our results to be consistent with those of Cheng et al. (2012), though [oxHb] may seem to respond more sensitively to BP changes than CBFV because phase changes were only marginally present in the BP-CBFV relationship.

Our study has limitations. Our CBF increases results are valid for movement tasks as the results are similar when movement is performed against a person’s own arm’s gravity or hand gripping at 30% of a person’s maximal hand gripping power (Caldas et al., 2018). Whether the dCA changes induced by movements are valid for changes in flow across the entire human cortex remains to be assessed since, for example, visual stimuli may provoke a different response pattern in the visual cortex compared to the motor cortex (Wolf et al., 2002). Further, our results are not independent of changes in ETCO_2_ and CVR. It appears necessary to at least control for CO_2_ changes in further studies. Nonetheless, the phase in the BP-[oxHb] system seems more promising than in the BP-CBFV system for our intended purpose of grading VNC.

The variances as indicated by the SDs in the NIRS parameter-dependent systems were large and corresponded to the findings of Cheng et al. (2012). Further data processing with the help of filtering might be helpful in reducing variance (Elting et al., 2018) but may lead to the loss of valuable information. Between-subject variability seems large and remains an unresolved problem (Sanders et al., 2018). One way to overcome subject variability is using a standardized examination setting and recording and reporting procedures (Claassen et al., 2016; Sanders et al., 2018). So far, our TFA results of the BP-CBFV system are consistent with those of a recent survey (Sanders et al., 2018). Another investigation could address whether using TF estimates as a strict linear method is adequate for assessing microcirculation. Aside from one study by Rowley et al. (2007), which discusses the presence of non-stationarity in the brain’s slow oscillation, few studies address [oxHb]- and [deoxHb]-dependent systems with regard to the presence of nonlinearity or non-stationarity.

In conclusion, in our experiment assessing elbow movements performed against gravity, we found an increase in cerebral blood flow in macro- and microcirculation, which was accompanied by a small but significant BP increase and a small but significant ETCO_2_ decrease. Despite vasoconstricting effects of BP increase and ETCOE decrease, the increase in the BP-CBFV system indicates vasodilation. With BP oscillations as the reference, elbow movement induced a significant delay in [oxHb] and [deoxHb] oscillations while leaving the transit time between [oxHb] and [deoxHb] unchanged. Microcirculatory changes in phase seem to be more convenient than macrocirculation responses when using complex physiological processes for grading the response of VNC.

## Data Availability Statement

The datasets generated for this study are available on request to the corresponding author.

## Ethics Statement

The studies involving human participants were reviewed and approved by Ethikkommission Nordwestschweiz. The patients/participants provided their written informed consent to participate in this study.

## Author Contributions

Both authors contributed equally to the work.

### Conflict of Interest

The authors declare that the research was conducted in the absence of any commercial or financial relationships that could be construed as a potential conflict of interest.
